# Case Report: Liraglutide for Weight Management in Beckwith-Wiedemann Syndromic Obesity

**DOI:** 10.3389/fendo.2021.687918

**Published:** 2021-06-22

**Authors:** Marina Caputo, Tommaso Daffara, Simonetta Bellone, Valentina Mancioppi, Paolo Marzullo, Gianluca Aimaretti, Flavia Prodam

**Affiliations:** ^1^ Endocrinology, Department of Translational Medicine, Università del Piemonte Orientale, Novara, Italy; ^2^ Department of Health Sciences, Università del Piemonte Orientale, Novara, Italy; ^3^ Division of Pediatrics, Department of Health Sciences, Università del Piemonte Orientale, Novara, Italy

**Keywords:** Beckwith-Wiedemann syndrome, liraglutide, syndromic obesity, obesity, weight

## Abstract

Genetic obesity, including syndromic and non-syndromic forms, represents a minority of cases compared to essential obesity but gene dysregulations lead to complex clinical conditions that make their management particularly difficult. Among them, Beckwith-Wiedemann syndrome (BWS) is a multisystem human genomic imprinting disorder characterized by overgrowth. We describe the first case of liraglutide treatment in an 18-year-old boy patient affected by BWS complicated by macroglossia, cryptorchidism, nephroblastoma, organomegaly, microscopic lymphocytic colitis, pharmacologically treated arterial hypertension, obesity, and obstructive sleep apnea syndrome. He presented a normal cognitive development. Body mass index at the time of first transition visit in the adult endocrinology department at the age of 18-years-old was 40.6 kg/m^2^ without glucose metabolism impairment. Lifestyle interventions failed because of poor compliance. During 20 months of 3.0 mg liraglutide treatment, a weight loss of 19 kg (−13.3%) and BMI reduction of 6.8 points were registered without side effects. To date, liraglutide treatment was effective on obesity in 7 subjects with Prader Willy Syndrome and 14 with melanocortin-4 receptor mutations. The efficacy of liraglutide in BWS could be related to a crosstalk among glucagon-like peptide (GLP)-1 system, mechanisms related to the cyclin-dependent kinase inhibitor 1C (CDKN1C), and dopamine mesolimbic circuit. Clinical trials aiming at a tailored medicine in genetic obesity are needed.

## Introduction

Obesity is a major public health problem (1) that leads to high personal, social, and economic costs of its related comorbidities (2). Although the major causes of obesity are obesogenic lifestyle habits, genetic forms, including syndromic (ORPHANET code 240371) and non-syndromic monogenic obesities (ORPHANET code 98267) should be taken into account to early recognize them thus activating proper management. Among genetic obesity, syndromic forms are often associated with an early-onset of severe obesity with specific neurodevelopmental phenotypes (3).

Beckwith-Wiedemann syndrome (BWS) is a multisystem human genomic imprinting disorder characterized by overgrowth with variable clinical phenotype (4) including early onset obesity (5).

The syndrome is caused by genetic and epigenetic changes on the chromosome 11p15 region, which includes genes such as cyclin-dependent kinase inhibitor 1C (CDKN1C) or insulin-like growth factor 2 (IGF-2), strongly related to fetal and postnatal growth. Genetic and epigenetic alterations are frequently mosaic and lead to a range of different clinical phenotypes; macroglossia, abdominal wall defects, hemihyperplasia, enlarged abdominal organs, hypoglycemia with hyperinsulinemia at birth, and an increased risk of embryonal tumors during early childhood are the most frequent manifestations. According to the last consensus statement, BWS could be considered as a spectrum of diseases. The estimated prevalence is different worldwide and ranged from 1:79,500 in Spain to 1:10,340 in Italy. Increased risk of BWS with assisted reproductive technologies has been reported (6, 7).

A clinical scoring system has been proposed to decide if molecular testing for BWS should be requested and to establish a clinical diagnosis of the syndrome. In this system, cardinal characteristics are macroglossia, omphalocele, lateralized overgrowth, bilateral Wilms tumor, hyperinsulinism, and specific pathological findings such as adrenal cytomegaly or placental mesenchymal dysplasia. Each of these features amounts to 2 points. Suggestive characteristics include birth weight greater than 2 standard deviations (SD) above the mean, facial nevus simplex, polyhydramnios or placentomegaly, ear creases or pits, transient hypoglycemia, embryonal tumors, nephromegaly or hepatomegaly, and umbilical hernia or diastasis recti. Each of these suggestive features amounts to 1 point. Patients with a score ≥ of 4 satisfy a clinical diagnosis of classical BWS, even without molecular confirmation. For patients with a clinical score ≥ of 2 genetic testing for BWS is considered. If a patient with a score ≥ of 2 presents negative genetic testing, alternative diagnoses and/or referral to a BWS expert should be taken into account. On the contrary, patients with a score <2 do not meet the criteria for genetic testing. Genetic testing is recommended also for patients with a family history of BWS and a known heritable pathogenic 11p15 anomaly, which occurs in about 10–15% of patients (7, 8).

Recently Barisic et al. (6) reported data from the European registry of surveillance of congenital anomalies (EUROCAT), showing that among patients with BWS, neonatal macrosomia was recorded in a range between 24.0 and 49.2%.

BWS patients can show early-onset obesity, although BWS is often an underestimated cause of syndromic obesity (5). Albeit relevant for patients and their families, little information regarding adulthood health status is available in the literature (9). In the first large cohort of adults BWS recently described (9), final height was up to +2 SDS in 44% of patients and 57.7% of cases showed height above their genetic target. Moreover, adult features were often related to complications of pediatric developmental defects and/or surgical interventions. Thus, the importance of early management of syndrome-related diseases is meaningful.

Here, we describe the first case of a young adult affected by BWS obesity without diabetes treated by a glucagon-like peptide-1receptor agonist (GLP-1 RA).

## Case Description

An 18-year-old boy affected by BWS transitioned from Pediatric Endocrinology to our Endocrinology Unit.

Prenatal diagnosis of BWS was made because of macrosomia, omphaloceles, and polyhydramnios, and an Imprinting Center 1 Gain of Methylation (GoM IC1) associated with IC1 microdeletion (10) was detected.

He was born at 30 weeks by cesarean delivery, with a birth weight of 4,500 g. The glucose levels at birth were within the normal range, without hypoglycemia.

At birth, he suffered from pneumothorax. At the age of 1 year old, he was surgically treated for macroglossia and left cryptorchidism. At the age of 5 years, nephroblastoma was diagnosed and treated by surgery and chemotherapy. He presented a normal cognitive development.

At the age of 12 years old, arterial hypertension was diagnosed. Regarding complications, the doppler ultrasound (US) examination of carotid arteries was normal, the electrocardiogram showed a right bundle branch block while the cardiac ultrasonography revealed minimal mitral insufficiency. Ramipril 5 mg *per day*, increased up to 10 mg, and adding amlodipine 5 mg *per day* for uncontrolled blood pressure values was started.

At the age of 16 years old, for recurrent otitis, he underwent surgical myringoplasty.

Recurrent abdominal pain and episodical rectorrhagia were investigated, and the microscopic colitis lymphocytic colitis subtype was diagnosed.

Over the years, lateral growth assessed up to the 90^th^ centile. Weight and Body Mass Index (BMI) were up to the 90^th^ percentile from birth and progressively increased; he was diagnosed obese at the age of 8.6 years with a BMI at 99^th^ centile (24.38 kg/m^2^). He was under nutritional treatment and physical activity program all life long, although unsuccessful. Obesity was complicated by obstructive sleep apnea syndrome (OSAS) diagnosed at the age of 13 years old, treated by non-invasive ventilation.

During the pediatric follow-up, glucose metabolism assessment was always normal; pituitary function was preserved with evidence of mild elevation of Insulin-like growth factor (IGF)-I.

At the time of the first transition visit, at the age of 18 years old, the patient height was 186 centimeters (cm) (96^th^ percentile; 1.8 SDS), up to his genetic target (166.7 cm), weight was 140.5 kilograms (kg) and Body mass index (BMI) was 40.6 kg/m2. Waist circumference measured 130 cm. The pubertal stage was at term. Blood pressure was 140/80 mmHg and heart rate 80 bpm under treatment. Ambulatory blood pressure monitoring (ABPM) showed borderline hypertension with reduced nocturnal dipping. US abdomen examination showed no sign of hepatomegaly or steatosis.

Renal and liver function were normal, with evidence of renal hyperfiltration (eGFR 124 ml/min/1.73m^2^) and compensatory hyperplasia of the remaining kidney, microalbuminuria was negative (2 mg/24 h); he was tumor-free.

The study of basal pituitary function (corticotropic, thyroid, gonadal, and somatotroph axes) was confirmed as normal.

The ongoing therapy was amlodipine 5 mg, ramipril 10 mg, ferrous sulfate 105 mg, cholecalciferol 25,000 UI/month, cetirizine 10 mg.

Fasting laboratory test results were notable for total cholesterol of 121 mg/dl, low-density lipoprotein cholesterol (LDL) of 64 mg/dl, triglycerides of 76 mg/dl, high-density lipoprotein (HDL) of 42 mg/dl, glycosylated hemoglobin (HbA1c) was 5.1%. Oral glucose tolerance test (OGTT at time 0-60-120 min) showed normal glucose (84-93-88 mg/dl) and insulin levels (16.5-33.2-22.9 µUI/ml) with moderate insulin resistance at fasting (HOMA-IR 3.42), and a normal Matsuda index (5.52). No hypoglycemic episodes were reported.

Regarding his weight history, diet intervention was unsuccessful although analysis of the food diary showed good compliance to diet regimen. After 3 months of follow-up, during a Mediterranean-style diet of 2,000 Kcal, tailored on his basal metabolism, weight increased up to 142 kg with a BMI of 41.1 kg/m^2^ because of hyperphagia. He followed an aerobic physical activity program of 30 min per day.

**Figure 1:**
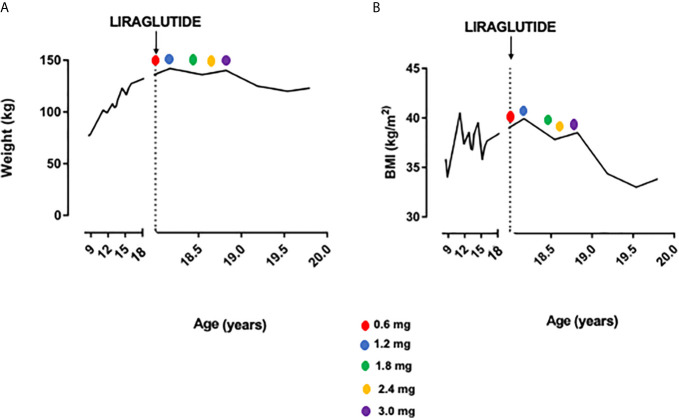
Weight **(A)** and BMI **(B)** trend before and after liraglutide treatment.

**Figure 2:**
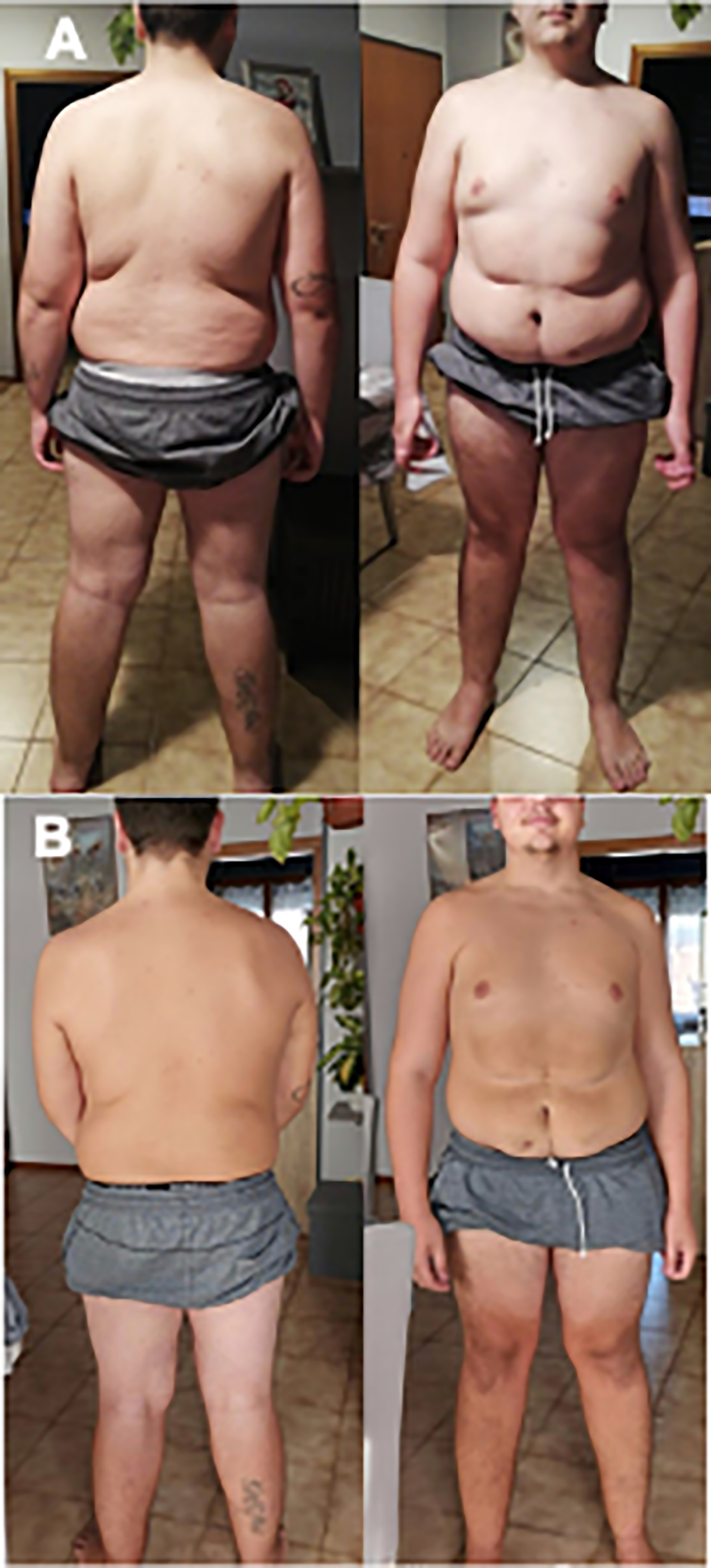
Pictures of patient before **(A)** and after **(B)** liraglutide treatment.

Thus, considering severe obesity complicated by blood hypertension and OSAS unresponsive to diet and lifestyle interventions, we proposed a GLP1-RA therapy with liraglutide. In November 2018, liraglutide 0.6 mg/day was started, increased up to 1.8 mg. After 6 months of follow-up, weight was reduced to 136 kg (BMI 37.8 kg/m^2^) without side effects. The dose was titrated up to 3.0 mg per day. In November 2019 weight was 125 kg (BMI 34.4 kg/m^2^), and at the last follow-up visit of July 2020 weight was 123 kg (BMI 33.8 kg/m^2^), with a total weight loss of 19 kg (−13.3%) and BMI reduction of 6.8 points in 20 months (shown in **Figures 1** and **2**). No side effects were registered.

The ambulatory blood pressure monitoring showed adequate diastolic and systolic levels and the ramipril dose was reduced to 5 mg per day.

Normal glycemic and metabolic pattern was documented (fasting glucose 92 mg/dl, HbA1c 5.0%, uric acid 4.0 mg/dl, total cholesterol 136 mg/dl, LDL 64 mg/dl, triglycerides 75 mg/dl, HDL 57 mg/dl). No hypoglycemic episodes were reported. IGF-I levels were in the lower part of the reference range (176.3 ng/ml).

The patient referred to amelioration of quality of life with more social interactions and he was motivated to begin a program of structured physical activity. He referred a decrease in hyperphagia, confirmed by the reduction of the score of visual analog scale with respect to baseline.

Clinical, anthropometric, and metabolic features during liraglutide treatment are summarized in **Table 1**.

**Table 1:** Clinical, anthropometric, and metabolic features at baseline and during liraglutide treatment.

Clinical, anthropometric, and metabolic features	Baseline	6 months	12 months	19 months
Age (years)	18	18.5	19	19.6
Liraglutide dose (mg/day)	0.6	1.8	3.0	3.0
Weight (kg)	142	136	125	123
BMI (kg/m^2^)	41.1	37.8	34.4	33.8
Side effects	–	no	no	no
Glucose (mg/dl)	84	–	92	–
HbA1c (%)	5.1	–	5.0	–
Total cholesterol (mg/dl)	121	–	136	–
HDL cholesterol (mg/dl)	42	–	57	–
Triglycerides (mg/dl)	76	–	75	–
LDL cholesterol (mg/dl)	63.8	–	64	–
IGF-I (ng/ml)	234.4	–	176.3	–

BMI, Body Mass Index; HbA1c, Glycosylated Hemoglobin; HDL, high density lipoprotein; IGF-I, insulin like growth factor I; LDL, low density lipoprotein.

## Discussion

We describe the first case of weight loss with GLP1-RA liraglutide in obesity caused by BWS.

GLP-1 is an anorexigenic hormone released from the L cells of the small intestine in response to nutrient intake. GLP-1 has several physiological functions, including decreasing food intake and increasing satiety and satiation (11, 12). The glucose-lowering properties of GLP1-RA together with proven cardiovascular and renal protective effects made this class of molecules a milestone in the treatment of patients affected by type 2 diabetes mellitus (T2DM).

Moreover, liraglutide has been approved for obesity treatment. Among pharmacological therapies for obesity, the GLP1-RA liraglutide was associated with the higher odds of weight loss of at least 5% and weight loss of at least 10% compared with orlistat, lorcaserin, and naltrexone-bupropion (13). Liraglutide once-daily dose of 3.0 mg in non-diabetic obese or overweight subjects in addition to a hypocaloric diet and increased physical activity was associated with increased weight loss with respect to placebo (−5 to −6 kg) with a demonstrated durability. Furthermore, it was associated with an improvement in waist circumference, blood pressure, inflammatory markers, and liver disease (14).

Less is known about the efficacy of GLP1-RA on syndromic obesity. To the best of our knowledge liraglutide effects on morbid obesity were described in 7 PWS patients all affected also by T2DM (15–18) and in a prospective case-control non-randomized study on 14 patients affected by monogenic obesity related to melanocortin-4 receptor (MC4R) mutations (19).

The characteristics of patients affected by PWS and treated by liraglutide are summarized in **Table 2**. All patients were under anti-hyperglycemic therapies and liraglutide at a maximum dosage of 1.8 mg per day. The starting of liraglutide resulted in a mean BMI and HbA1c reduction of 1.8 and 1.3 points, respectively. Further results are expected by an ongoing trial on pediatric PWS without T2DM (20).

**Table 2:** Demographic, clinical, and metabolic characteristics of PWS patients treated by liraglutide.

Author, year	Age (year)	Sex	LIRA dose(mg)	BMI baseline (kg/m²)	BMI control 1 (time from baseline)	BMI control 2 (time from baseline)	DIAB	DIAB treatment	HbA1c baseline (%)	HbA1c control 1 (time from baseline)	HbA1c control 2 (time from baseline	Endocrine deficiency
Fintini et al., 2014 (15)	37.3	M	1.2	36	33 (12 months)	32.4 (24 months)	Yes	MET 1700 mg	7.6	6.3 (12 months)	6.9 (24 months)	TST
Fintini et al., 2014 (15)	27.7	M	1.2	44	44 (12 months)	44 (24 months)	Yes	MET 1700 mg	7.5	6.9 (12 months)	7.4 (24 months)	No
Fintini et al., 2014 (15)	30.4	F	1.2	50	48 (12 months)	48 (24 months)	Yes	MET 3000 mg	8.7	7.3 (12 months)	7.8 (24 months)	No
Fintini et al., 2014 (15)	37.1	F	1.8	30	31 (12 months)	30.2 (24 months)	Yes	MET 2000 mgGliclazide 30 mg	8.3	8.6 (12 months)	9.3 (24 months)	Gonads
Kim et al., 2020 (16)	18	F	1.2	59.8	50.8 (3 months)	48.5 (12 months)	Yes	MET 1500 mgLevemir	7.3	4.8 (3 months)	7.3 (12 months)	GH, gonads
Senda et al., 2012 (17)	25	F	0.9	39.1	35.7 (12 months)		Yes	/	12.6	6.1 (12 months)		Unknown
Cyganek et al., 2011 (18)	18	F	1.8	65.2	−2 kg (8 weeks)	−3.2 kg (14 weeks)	Yes	MET 1500 mg	8.5	6.7 (8 weeks)	6.6 (14 weeks)	GH

BMI, Body Mass Index; DIAB, Diabetes Mellitus; GH, Growth Hormone; HbA1c, Glycosylated Hemoglobin; LIRA, Liraglutide; MET, Metformin; TST, testosterone.

To date, the only study that assessed the effect of 3.0 mg liraglutide for the treatment of monogenic obesity without hyperglycemia has been conducted in adult patients affected by MC4R pathogenic mutations. A 16-week treatment in 14 subjects cases matched with 28 controls with non-syndromic obesity resulted in a weight loss of 6% in both groups, suggesting that the molecule can also be pro-satietogenic in MC4R- linked obesity (19).

These results are in line with our BWS patient. Liraglutide titrated up to 3.0 mg resulted in a reduction of weight of 19 kg with a 13.3% weight-loss and BMI reduction of 6.8 points, more than that theoretically expected based on experiences on patients with MC4R mutations or PWS.

A possible explanation of this phenomenon is the relationship between CDKN1C function and the GLP-1 effect on the brain. CDKN1C is a member of the cyclin-dependent kinase inhibitors family (21, 22) and plays a key role in neurogenesis, migration, and morphology of the central nervous system (23–26), particularly promoting proliferation and differentiation of midbrain dopaminergic neurons (24). The role of CDKN1C in behavioral symptoms specifically related to food reward and hedonic responses to palatable foods has been demonstrated in imprinting disorders (27). Elevated expression of CDKN1C equivalent to a model of loss-of-imprinting, results in an altered dopaminergic neural state and increased motivation for reward and social dominance (27, 28). These findings are intriguing since liraglutide acts on satiety and suppresses food intake at central levels, apart from its pancreatic and gastric effects (29). Neuronal GLP-1 receptors have been discovered in the hypothalamus, striatum, midbrain, and hindbrain of primates (30) and humans (31) and GLP-1 has been found to act on the mesolimbic dopamine system of mice (32). Thus, we can speculate that the brain effects of liraglutide could support the result on weight loss and behavior in BWS in which the mechanism of food intake gratifications is amplified by gene dysregulations. CDKN1C hyperexpression results in altered dopaminergic tone and excessive food intake related to hedonic response to palatable foods. Neuronal GLP1-R activation by liraglutide could restore a normal dopaminergic state, hence reducing food consumption and, as consequence, body weight. These speculations should be taken into account for implications and management of other imprinting disorders associated with altered CDKN1C (33).

The causative mutation of BWS could interest genes such as IGF-2, implicated in postnatal overgrowth. Interestingly, the adipose tissue expresses both IGFI-2 and its receptor together with GLP-1 receptors (34). Thus, considering that GLP-1 promotes preadipocyte differentiation, reduces the expression of adipogenic and lipogenic genes, and enhances the expression of lipolytic markers (35), these could be some mechanisms targeted by liraglutide in BWS. Of note, IGF-2 levels were negatively correlated with visceral adipose tissue (VAT) in a cohort of patient with T2DM or prediabetes; treatment with liraglutide was associated with increased IGF-2 and reduction of VAT, establishing a potential correlation between IGF-2 and liraglutide mechanisms and supporting our hypothesis in BWS (36). Moreover, GLP-1 stimulates IGF1 receptor expression and Akt phosphorylation, and activation of IGF-1 receptor signaling is dependent on the secretion of IGF-2 in the called “IGF-2/IGF-1-receptor autocrine loop” (37); this cross-talk showed a protective role on the beta cell in mice (37), that could enhance GLP-1RA effects in BWS patients.

**Figure 3:**
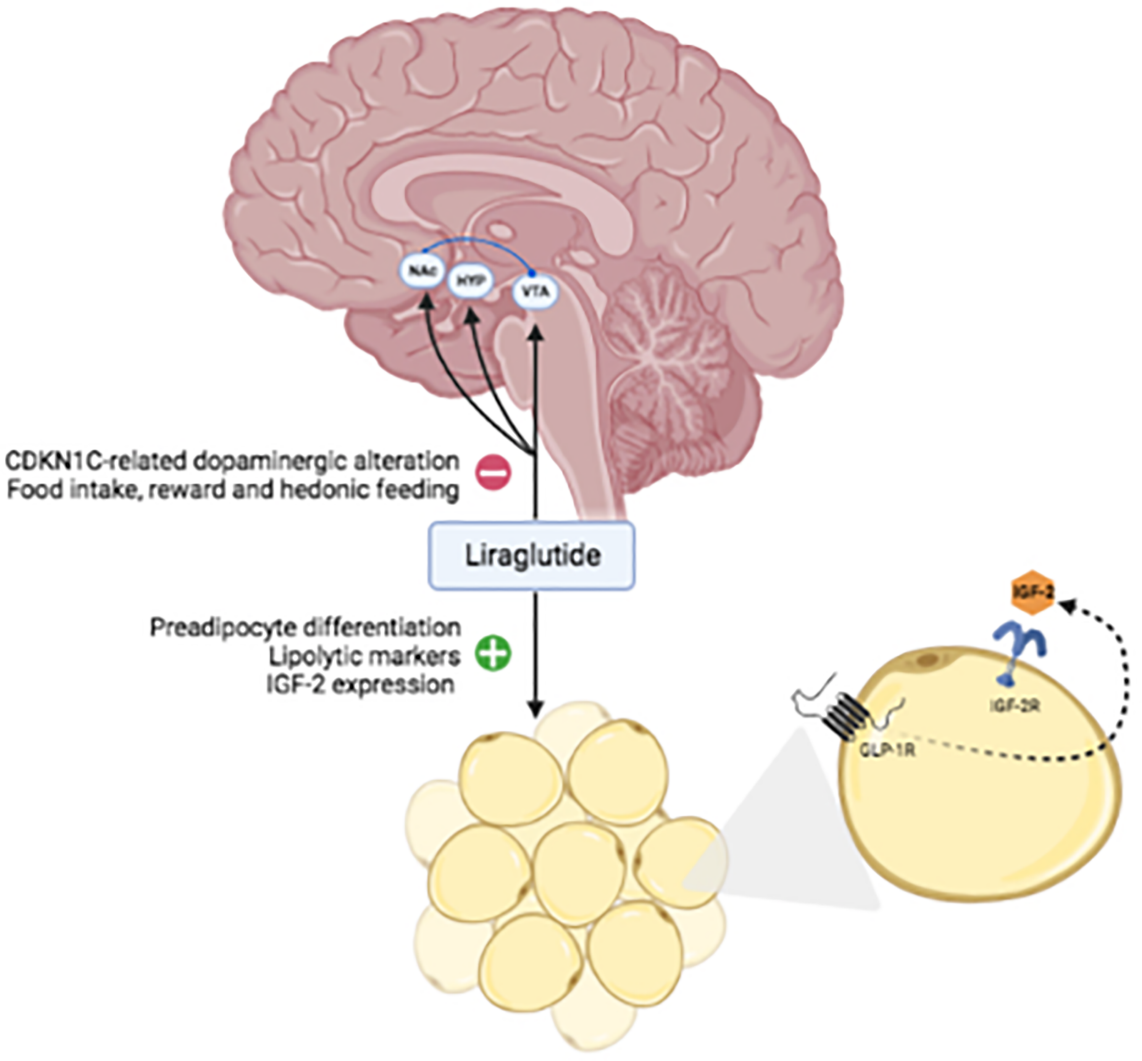
Hypothetical mechanisms targeted by liraglutide in BWS. Abbreviations: VTA, ventral tegmental area; NAc, nucleus accumbens; HYP, hypothalamus; IGF, insulin-like growth factor; IGF-2R, insulin-like growth factor 2 receptor; GLP1-R, glucagon-like peptide1-receptor.

Thus, the effect of liraglutide on body weight in patients with BWS could be related to an effect on the central nervous system and a peripheral effect on adipose tissue (**Figure 3**). Studies on animal models are necessary to validate this hypothesis.

Moreover, among the other extra-hypoglycemic effect of liraglutide, inhibition of proliferation and promotion of apoptosis in tumoral cell lines has been demonstrated (38). This effect should be particularly important in BWS patients, who are at higher tumoral risk.

A critical point in this context is the risk of hypoglycemia, which occurs in at least 50% of neonates with BWS, caused by hyperinsulinism (39). In most cases hypoglycemia is transient but up to 20% of patients may present persistent forms that could require pancreatectomy. In our patient, a normal gluco-insulinemic metabolism was assessed during his lifespan, but the risk of hypoglycemia, although low during liraglutide treatment, should be taken into account considering the intrinsic predisposition in BWS patients.

Patients with genetic obesity need a comprehensive approach considering the complexity of their clinical conditions. Considering the eating behavior previously described, dietary interventions are intuitively going to fail. Thus, a multidisciplinary approach by an expert team is essential to improve long-term outcomes in these patients (3). Tailored therapy is necessary for genetic obesity. This is the first treated BWS subject with liraglutide, being a proof-of-concept study but we also recognize the intrinsic limitation due to the heterogeneous genetic background of the BWS. Further studies in BWS patients complicated by obesity are required to confirm our results and assess whether patients with different genetic mutations present the same effects after liraglutide treatment.

## Data Availability Statement

The original contributions presented in the study are included in the article/supplementary material. Further inquiries can be directed to the corresponding author.

## Ethics Statement

Written informed consent was obtained from the individual for the publication of any potentially identifiable images or data included in this article.

## Author Contributions

MC, TD, and VM collected the anamnestic as well as the biochemical data for the patient. MC and TD performed the literature search, reviewed and extracted data from the papers. MC, TD, and FP performed the data analysis, the figures and tables designing, and the manuscript writing. FP, SB, PM, and GA verified the analytical methods and supervised the manuscript drafting. All authors discussed the results. All authors contributed to the article and approved the submitted version.

## Conflict of Interest

The authors declare that the research was conducted in the absence of any commercial or financial relationships that could be construed as a potential conflict of interest.
